# Sternectomy for *Candida albicans* sternal osteomyelitis after left ventricular assist device implantation

**DOI:** 10.1007/s12055-021-01144-x

**Published:** 2021-02-17

**Authors:** Mathias Van Hemelrijck, Michelle Frank, Annelies S. Zinkernagel, Ronny Buechel, Juri Sromicki, Markus J. Wilhelm, Holger Klein, Barbara Hasse, Carlos A. Mestres

**Affiliations:** 1 Clinic for Cardiac Surgery, University Hospital Zurich, Rämistrasse 100, CH-8091 Zürich, Switzerland; 2 Clinic for Cardiology, University Hospital Zurich, Zurich, Switzerland; 3 Division of Infectious Diseases and Hospital Epidemiology, University Hospital Zurich, Zurich, Switzerland; 4 Department of Nuclear Medicine, Cardiac Imaging, University Hospital Zurich, Zurich, Switzerland; 5 Department of Plastic Surgery and Hand Surgery, University Hospital Zurich, Zurich, Switzerland

**Keywords:** Left ventricular assist device, Endocarditis, Sternectomy, Fungal osteomyelitis

## Abstract

Fungal osteomyelitis is an uncommon complication after cardiac surgery and associated with high mortality. A case of *Candida albicans* and *Staphylococcus epidermidis* osteomyelitis with device infection after implantation of a left ventricular assist device in a 60-year-old male patient is presented here. After clinical identification and confirmation with microbiological examinations and fluorodeoxyglucose positron emission tomography (FDG-PET) scan, debridement was performed. Surgical specimens grew *C. albicans* and *S. epidermidis*. Fluconazole, daptomycin, and negative pressure wound therapy were initiated, but failed to achieve healing. Total sternectomy and pectoralis flap reconstruction were performed. There was no recurrent infection for *C. albicans* on a prolonged antifungal regime. The combination of antifungal therapy and aggressive surgical debridement may be useful to control fungal osteomyelitis.

## Introduction

Fungal osteomyelitis is a serious albeit uncommon condition after cardiac surgery. It requires aggressive therapy and is associated with high mortality. Treatment strategy includes prolonged antifungal therapy and surgical debridement, whereas the extent of excision is still a matter of debate. A case of *Candida albicans* and coagulase-negative staphylococci sternal osteomyelitis and concomitant left ventricular assist device (LVAD) infection, its therapy, and outcome are discussed.

### Case report

**Fig. 1:**
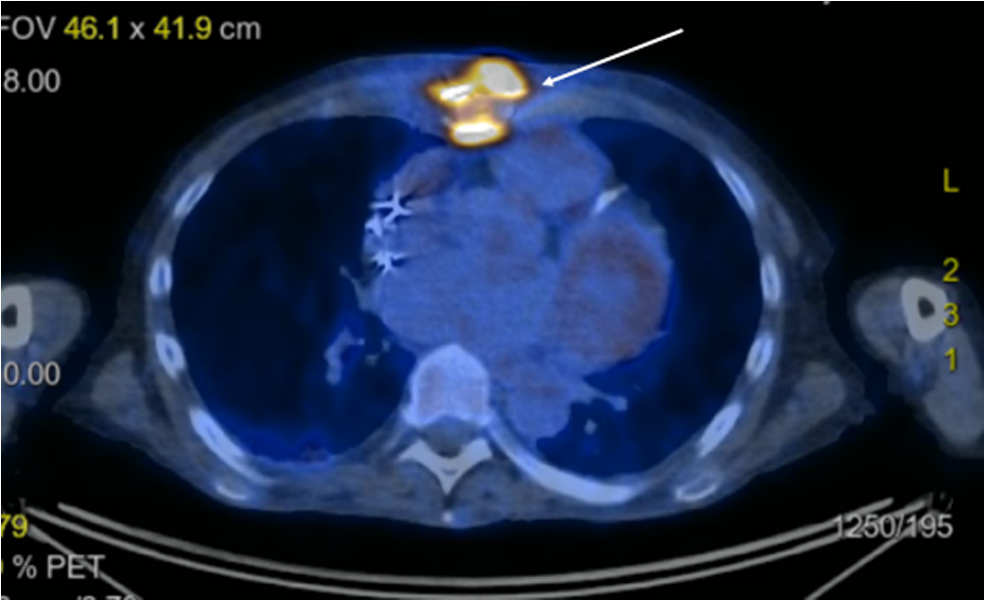
Sternal uptake in a fluorodeoxyglucose positron emission tomography (FDG-PET) scan

**Fig. 2:**
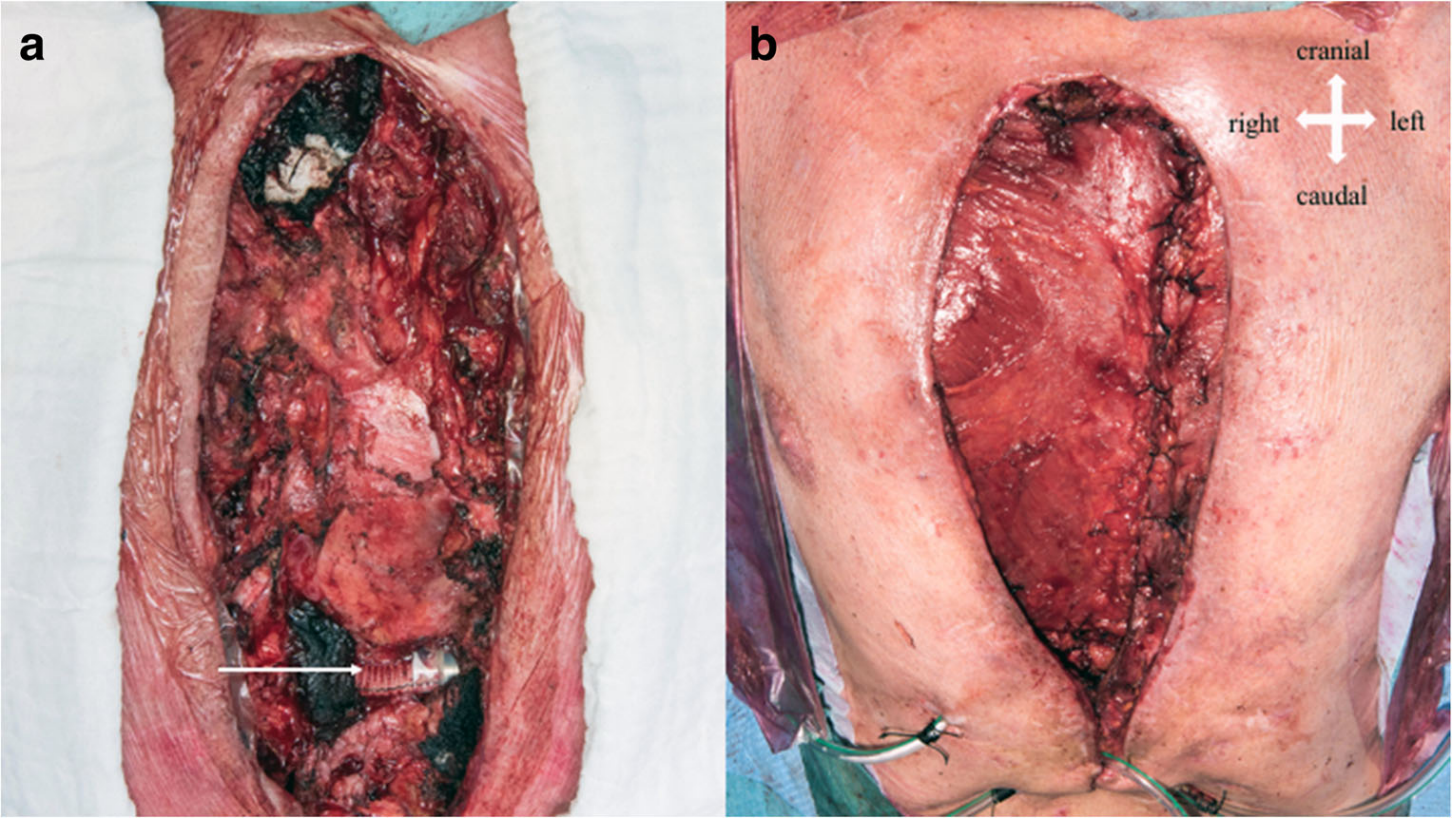
**a** Mediastinum after sternectomy: the arrow points to the caudal portion of the outflow cannula. **b** Pectoralis flap covering the mediastinum

A 60-year-old male patient underwent LVAD (HeartWare®) implantation in November 2017 as a bridge-to-transplantation due to ischemic cardiomyopathy. He required 6 mediastinal re-explorations due to recurrent bleeding. The revision procedures performed in this patient were always categorized by the operating surgeons as diffuse bleeding without surgical origin. The patient was given multiple coagulation factors, including Factor VIII, due to the suspicion of LVAD-associated von Willebrand disease. After bleeding was controlled, he subsequently was discharged from the hospital 2 months postoperatively. Three months later, he was readmitted due to pulmonary decompensation and sternal wound infection (SWI). Fluorodeoxyglucose positron emission tomography (FDG-PET) scan confirmed sternal uptake; the full report confirmed high suspicion of strongly metabolically active sternal infection with small retrosternal abscess formation. There was mild reactive lymphadenopathy, mediastinal and axillary on both sides, and only moderately increased metabolic activity around the LVAD, which could be due to artifacts. There were slightly metabolically active infiltrates in the dorso-basal areas of the right lower lobe with suspected fibrotic changes after pneumonia. Otherwise, no further infection foci were detected (Fig. 1). Surgical debridement of the sternum was performed. *C. albicans* and *Staphylococcus epidermidis* grew in the operative samples. Attention was then paid to the sternal region as per clinical signs and FDG-PET information as described. Negative pressure wound therapy (NPWT) was initiated. NPWT lasted 3 months, until the wound was closed. The antimicrobial therapy included caspofungin 50 mg/24 h IV and daptomycin 10 mg/kg body weight/24 h IV. No attempt to work with antifungal and or antibiotic-impregnated crystals/granules was made, due to the lack of supporting evidence. Sequential samples of sternal tissue, taken during repetitive wound revisions, were persistently positive for *C. albicans* and *S. epidermidis*. Blood cultures were negative, and a subsequent FDG-PET scan suggested persistent sternal infection and a new abscess-like formation around the LVAD. We scheduled the patient for surgical resection and dead space filling with an adjacent muscle flap. LVAD exchange was not an option, since the new device would have had to be implanted in an already infected area. Heart transplantation could not be offered to the patient due to his poor condition. LVAD explantation was not an option because of severely reduced left ventricular function. Total sternectomy and pectoralis muscle flap closure of the chest were successfully performed (Fig. 2). After a complicated in-hospital course, which included pneumonia requiring temporary veno-venous extracorporeal membrane oxygenation (ECMO) and add-on of an antimicrobial therapy with meropenem 2 × 1 g/day i.v. (nosocomial pneumonia) and clarithromycin 2 × 500 mg/day p.o. No LVAD dysfunction was noticed at any time after sternectomy and muscle-plasty chest closure. The patient was discharged after almost 9 months in the hospital.

**Fig. 3:**
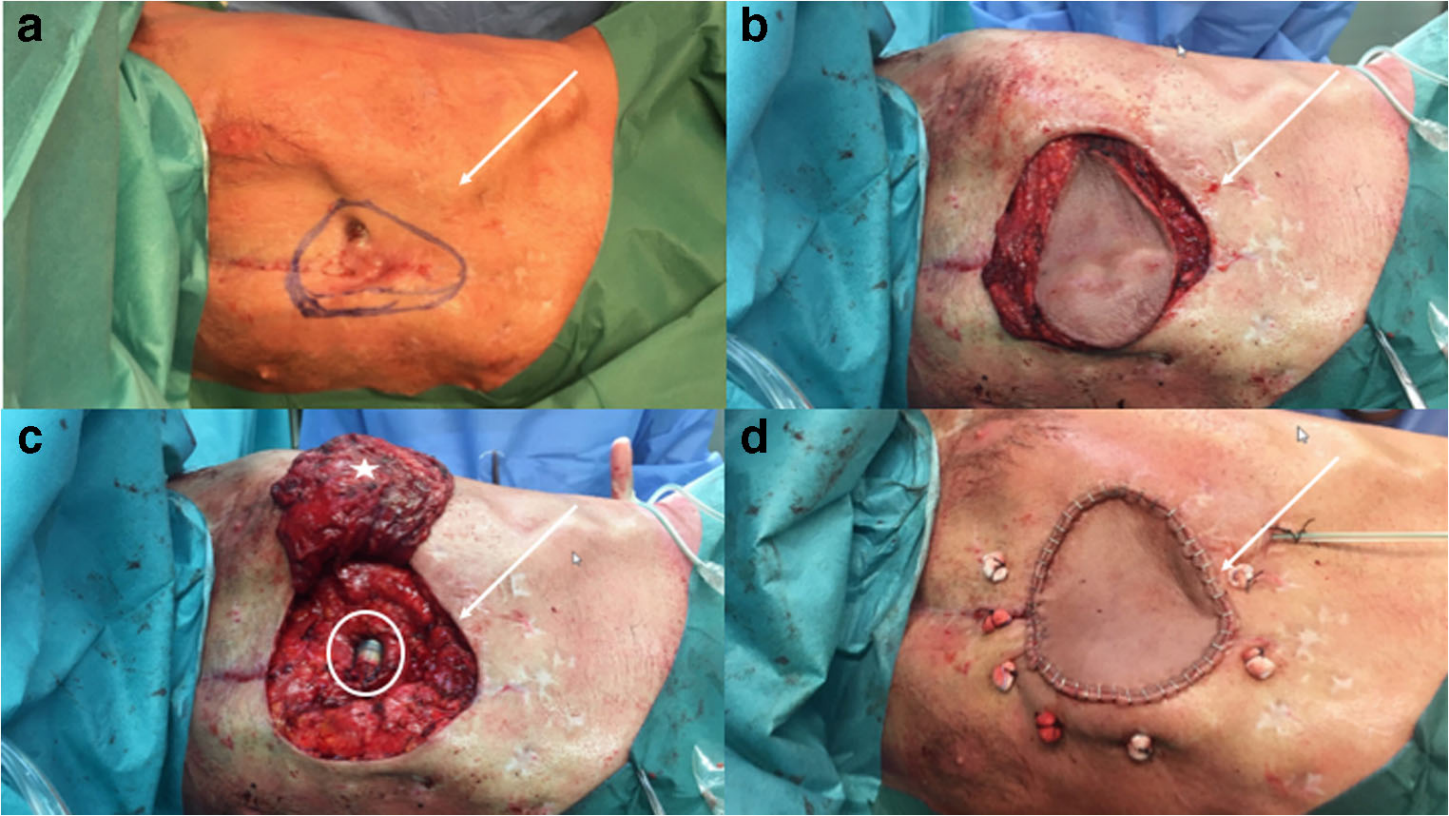
Latissimus dorsi muscle flap: **a** before incision, **b** after skin demarcation; **c** before covering the mediastinum and left ventricular assist device (circle) with a latissimus dorsi muscle flap (star); **d** final result. The arrow points in all images to the mediastinum

Six months later, under suppressive antifungal therapy with oral fluconazole 400 mg/24 h, no signs of fungal growth could be observed. However, a mechanical skin perforation above the LVAD outflow cannula was documented and subsequent debridement with LVAD coverage by a myo-cutaneous latissimus dorsi muscle flap was performed (Fig. 3). Cultures of the excised ulcerated tissue and all collected blood cultures did not show fungal or antimicrobial growth; furthermore, aspergillus antigen was negative (index 0.04). After an initially satisfactory postoperative course, the patient developed bilateral pneumonia and died 9 days after flap coverage. Postmortem examination disclosed respiratory failure with diffuse alveolar damage as the immediate cause of death. The pathological examination confirmed onset of acute bronchopneumonia of the right lower lobe. No pathogen was detected in special and immunohistochemical stains, especially for fungi or viruses. Numerous iron-laden intra-alveolar macrophages; fibrosis demarcated pleural empyema on the left (12 × 6 × 6 cm); trachea with subglottic scarring (max. 1.3 cm, probably post-intubation); pleural adhesions on both sides; and fibrinous pleurisy with no pleural effusion were noticed. In essence, further findings showed a chronic left-sided pleural empyema with pus surrounding the LVAD driveline. However, there were no persistent signs of osteomyelitis. Summarizing, the first revision for deep sternal wound infection (DSWI) was performed 6 months after LVAD implantation. Sternectomy was finally performed 5 months after the first debridement and 11 months after LVAD implantation. Overall, the patient died from hospital-acquired pneumonia 20 months after the LVAD was implanted.

### Comment

Deep sternal wound infection after cardiac surgery has an incidence of around 2% [1], with a reported mortality of 55% [2]. *Candida* osteomyelitis represents a severe and uncommon condition that requires combined surgical debridement and prolonged antifungal treatment. Re-sternotomies due to non-infective causes, prolonged use of antibiotics, colonization of the respiratory and urinary tracts, and the use of percutaneous dilatational tracheostomy devices have been identified as risk factors facilitating *Candida* infections [2]. Fungal osteomyelitis represents a treatment challenge, for which a number of surgical strategies have been suggested [1–3]. The optimal treatment strategy is still not clear [2, 3]. There are no significant outcome differences between the different surgical approaches, although more promising results have been confirmed with omental flaps in a recent review by Abu-Omar and colleagues [3]. But omental flaps require the opening of the abdominal cavity, harshly increasing the patient’s mortality. The use of a NPWT and a muscle flap is recommended by the European Association of Cardiothoracic Surgery (EACTS) as class I and IIb recommendation, respectively, with a level of evidence B in DSWI. However, there is no clear treatment strategy regarding fungal osteomyelitis [3]. On the other hand, Pappas et al. recommend surgical debridement and an antifungal regime of 6 to 12 months [4]. These recommendations are based on case reports and small series [5, 6]. In terms of timing of secondary wound closure, there is still a gap of knowledge among current guidelines and recommendation reports.

The implicated patient suffered from a combination of DSWI and LVAD-associated infection. In a recent multicenter study, Tattevin and colleagues reported 30% of infections after LVAD implantation, with 4% being cannula or pump related. *Candida-*associated LVAD infections were found in 6% of the patients. In this prospective study, the mortality rate among infected patients was 10% [7]. Regarding treatment strategies, it has been suggested that persistent infections under a suppressive antibiotic regime might require device explantation and subsequent heart transplantation [8]. In the case reported herein, an initial debridement with NPWT was unsuccessful. The patient therefore underwent total sternectomy and muscle flap closure. Since neither device explantation nor heart-transplantation was possible in our case, we decided that long-term suppressive antifungal therapy was mandatory. We achieved control of fungal osteomyelitis in this exceedingly uncommon case of infection in a patient under long-term mechanical circulatory support.
